# A rare case of acute promyelocytic leukemia with ider(17)(q10)t(15;17)(q22;q21) and favorable outcome

**DOI:** 10.1186/s13039-020-00479-1

**Published:** 2020-04-10

**Authors:** Yongming Liu, Junqing Xu, Lina Chu, Limei Yu, Yanhong Zhang, Li Ma, Weihua Wang, Yangyang Zhang, Yimin Xu, Riming Liu

**Affiliations:** 1 Clinical Laboratory, Qindao University Medical College Affiliated Yantai Yuhuangding Hospital, No. 20, Yuhuangding East Road, Yan Tai, 264000 China; 2 Department of Hematological, Qindao University Medical College Affiliated Yantai Yuhuangding Hospital, No. 20, Yuhuangding East Road, Yan Tai, 264000 China

**Keywords:** FISH, RT-PCR, APL, *PML-RARA*, Ider(17)(q10)t(15, 17)(q22, q21)

## Abstract

**Background:**

Chromosomal rearrangements in addition to t(15;17) have been reported in 25-40% of APL patients, with a large predominance of trisomy 8. Other abnormalities are far less frequent, particularly as ider(17), and the prognostic significance is still unclear.

**Case presentation:**

We present the case of a patient with t(15;17)(q22;q21), der(15)t(15;17) and ider(17)(q10)t(15;17)(q22;q21). In particular, the RT-PCR result for *PML-RARA* of this patient was a false negative and mutational analysis of AML-related genes showed SNP rs2454206 in the *TET2* gene and yielded negative findings in other genes including *AML1*, *ASXL1*, *CEBPA*, *DNMT3A*, *FLT3*, *KIT*, *NPM1*, *TP53*, and *U2AF1*. After the early usage of arsenic trioxide combinated with ATRA and vigorous supportive treatment to maintain PLT ≥30×10^9^/L and FIB >1500 mg/L, this patient was under MMR and HCR without any clinical symptoms or signs until now.

**Conclusion:**

False negative reslults of RT-PCR analysis for *PML-RARA* are rare in APL and ider(17) is even more infrequent. To our knowledge, this is the first reported case of APL with ider(17) and false negative RT-PCR analysis results. The role of ider(17) in APL is still an ongoing investigation and limited by the small number of published cases. The patient reported here benefited from vigorous supportive treatment during the combination of ATRA and arsenic trioxide in induction chemotherapy and the clinical outcome was favorable.

## Background

Acute promyelocytic leukemia (APL) is a subtype of acute myeloid leukemia (AML) with specific clinical and biological features. APL is identified by distinctive morphology and characterized by the specific chromosomal rearrangement t (15;17) (q22; q21), resulting in the fusion of the retinoic acid alpha gene (RARA) on 17q21 to the promyelocytic leukemia gene (PML) on 15q22 [1]. The disease used to be one of the most fatal forms of acute leukemia with poor outcomes. The introduction of all-trans retinoic acid (ATRA) in combination with arsenic trioxide and other chemotherapies has resulted in complete remission rates >90% and long-term remission rates above 80% [2]. However, there is still a small population of APL patients who have a poor prognosis [3–5]. To the best of our knowledge, specific cytogenetic abnormalities can cause a change in treatment response, relapse and clinicopathological characteristics [6]. Chromosomal rearrangements in addition to t(15;17) have been reported in 25–40% of APL patients, with a large predominance of trisomy 8. Other abnormalities are far less frequent, particularly ider(17). The prognostic significance of these abnormalities is still unclear [3, 7]. Here, we present the case of a patient with t(15;17)(q22;q21), der(15)t(15;17) and ider(17)(q10)t(15;17)(q22;q21) identified by conventional cytogenetics study and dual-color dual-fusion fluorescence in situ hybridization, and dual-color break apart fluorescence in situ hybridization methods.

## Case presentation

**Fig. 1:**
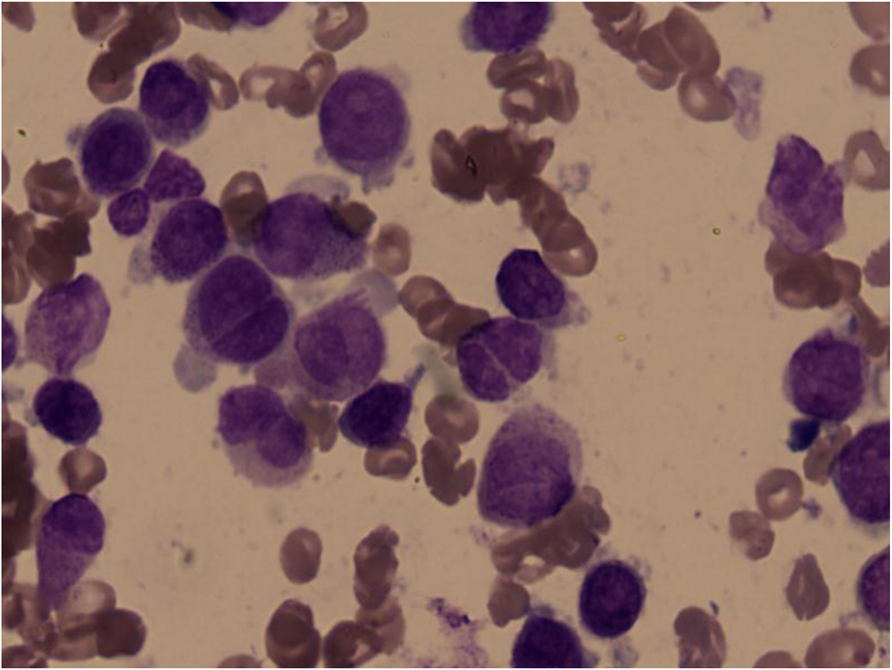
Bone marrow morphology at initial diagnosis. The bone marrow aspirate showed hypercellular marrow with increased abnormal promyelocytes, which had numerous granules, increased Auer rods, and visible of round or oval, distorted, folded nucleus

**Fig. 2:**
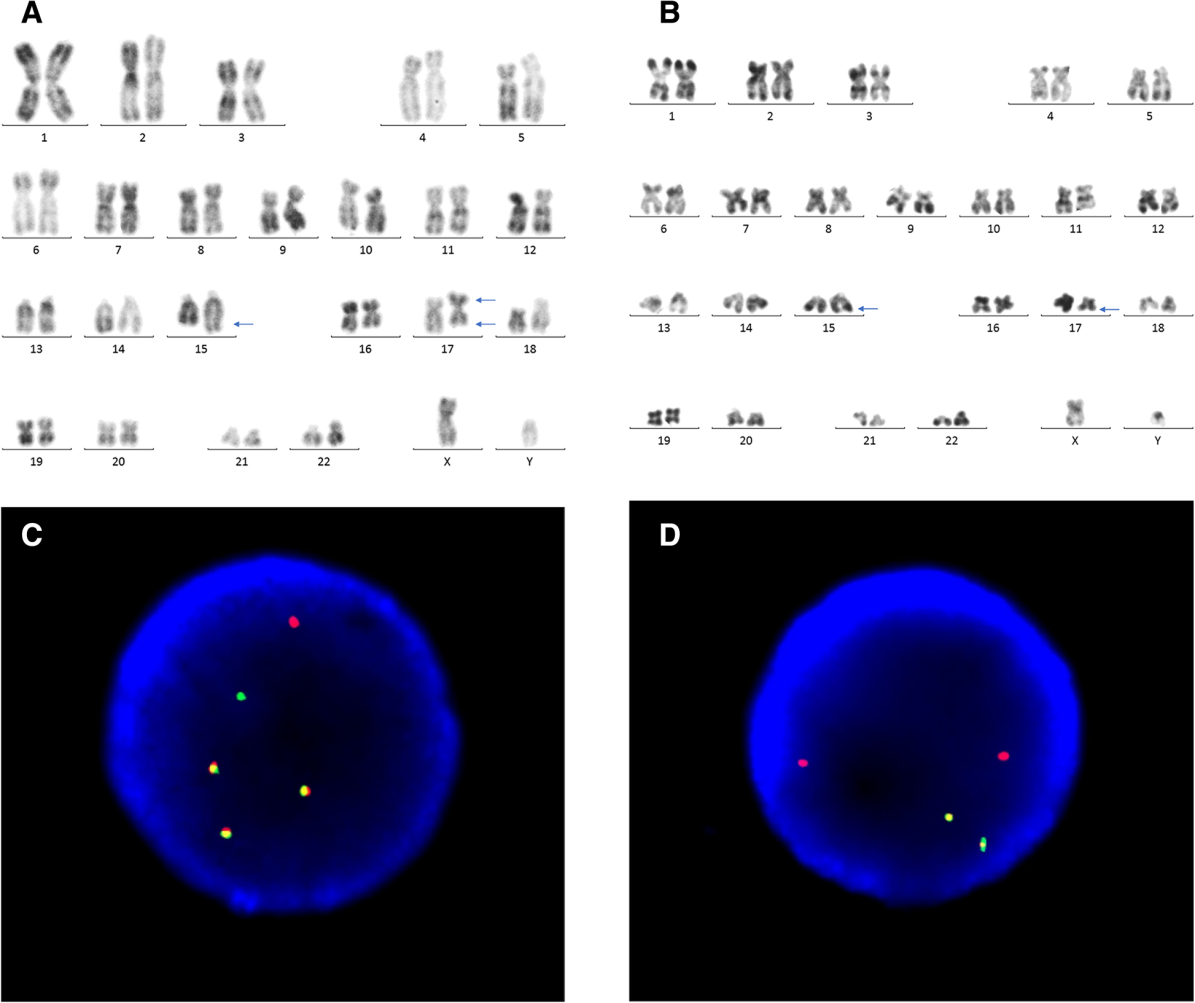
The karytyping and FISH result at initial diagnosis. FIGURE 2 (**a**): the karyotyping of 46,XY,der(15)t(15;17)(q22;q21),ider(17)(q10)t(15;17)(q22;q21). The arrows indicate abnormal chromosomes. FIGURE 2 (**b**): the karyotyping of 46,XY,t(15;17)(q22;q21). FIGURE 2 (**c**): FISH study using a PML-RARA dual-color, dual-fusion translocation probe. The metaphase cell showing three yellow *PML-RARA* fusion signals, one red signal (*PML*), and one green signal (*RARA*), consistent with the karyotype of ider(17)(q10)t(15;17)(q22;q21). FIGURE 2 (**d**): FISH study using a RARA dual color break apart rearrangement probe. The metaphase cell showing two red signals (5’*RARA*), one yellow fusion signal (*RARA*), and one green signal (3’*RARA*), consistent with the karyotype of ider(17)(q10)t(15;17)(q22;q21)

**Fig. 3:**
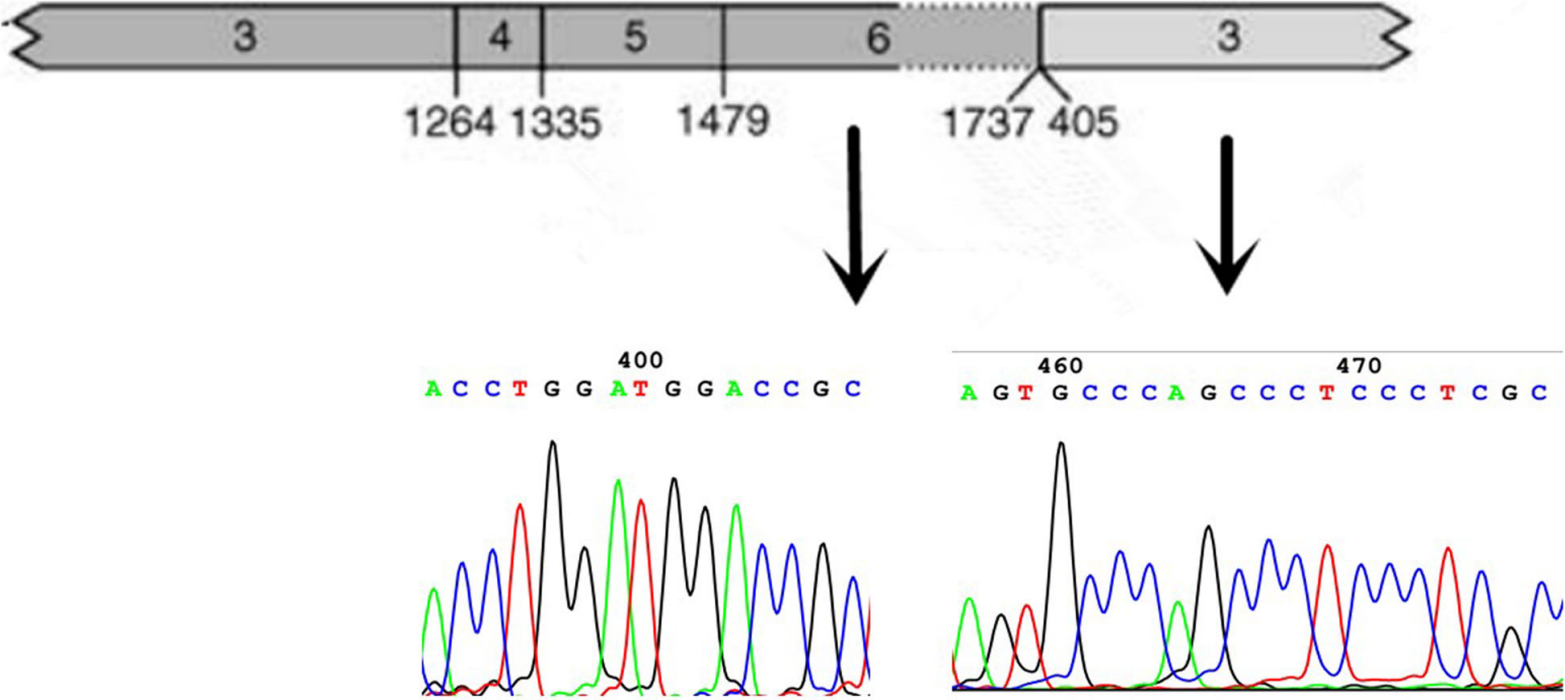
Sequencing analysis of *PML-RARA* fusion transcripts at initial diagnosis. Diagrammatic representation and sequencing information of *PML-RARA* fusion transcripts of the patient. *PML* transcripts consisting of exon 6 joined to *RARA* intron 3 (variant form)

In December 2018, a 26-year-old man was brought to Yantai Yuhuangding Hospital with the chief complaint of periumbilical pain and bleeding gums for 7 days. He had a fever of 38.7 Celsius. The initial complete blood count showed a hemoglobin level of 65 g/L, platelet count of 12 ×10^9^/L, and white blood cell count of 4.03 ×10^9^9/L with 59% promyelocytes, 6% neutrophils, 34% lymphocytes, and 1% neutrophilic metamyelocytes. Coagulation tests revealed that the prothrombin time, thrombin time and activated partial thromboplastin time were within the normal ranges, but the fibrinogen and D-dimer levels increased to 4.52 g/L and 20.77 mg/L, respectively. The bone marrow aspirate showed hypercellular marrow with 83% promyelocytes that had numerous granules, increased Auer rods, and strong myeloperoxidase activity (Fig. 1). Flow cytometry analysis of the bone marrow showed that 81.1% of blasts were strongly positive for CD38dim, CD13, myeloperoxidase, CD123, CD33str, and CD117, with partial expression of CD9, CD19, CD64, cCD79a and CD11b, whereas CD34, CD7, HLA-DR, CD36, CD56, CD4, CD14, CD15, CD16, CD71, CD2, CD5, CD105, CD20, mCD3, CD8, CD10, and cCD3 were negative. Mutational analysis of AML related genes using a high-throughput DNA sequencing technique showed the single nucleotide polymorphism (SNP) rs2454206 in the *TET2* gene (*TET2*:NM _−_001127208:exon11:c.A5284G:p.I1762V rs2454206, 48.28%) and yielded negative findings in other genes including *AML1*, *ASXL1*, *CEBPA*, *DNMT3A*, *FLT3*, *KIT*, *NPM1*, *TP53*, and *U2AF1*. Classical cytogenetic analysis showed 17 metaphase cells with 46,XY,der(15)t(15;17)(q22;q21),ider(17)(q10)t(15;17)(q22;q21); 1 metaphase cell with 46,XY,t(15;17)(q22;q21); and 2 normal cells according to ISCN2016 [8] (Fig. 2a and b). Fluorescence in situ hybridization (FISH) using a Vysis LSI *PML/RARA* dual color, dual fusion translocation probe (Abbott Molecular, Des Plaines, IL) indicated nuc ish (*PML* ×4), (*RARA* ×4), (*PML* con *RARA* ×3)[400/500], (*PML* ×3), (*RARA* ×3), and (*PML* con *RARA* ×2)[25/500] (Fig. 2c). FISH using a Vysis LSI *RARA* dual color break apart rearrangement probe revealed nuc ish (5’*RARA* ×4), (3’*RARA* ×2), (5’*RARA* con5’*RARA* ×2)[400/500], (5’*RARA* ×2), (3’*RARA* ×2), (5’*RARA* con5’*RARA* ×1)[25/500] (Fig. 2d), which is consistent with the result of FISH using the *PML/RARA* dual color, dual fusion translocation probe. Reverse transcriptase-polymerase chain reaction (RT-PCR) analysis for the *PML-RARA* rearrangement unexpectedly showed a false negative result; therefore, sequence analysis of *PML-RARA* were conducted. Sequencing of this sample confirmed that there was a fusion between *PML* exon 6 and *RARA* intron 3 (variant form)(Fig. 3). According to the sequence analysis results, new probes were designed, and the RT-PCR analysis for the *PML-RARA* rearrangement showed a positive result. This patient was diagnosed with APL according to bone marrow morphology, immunophenotyping, and molecular biology studies. He was treated with ATRA at a dose of 25mg/m2 per day and arsenic trioxide (As_2_O_3_) at a dose of 0.16 mg/kg per day for 30 days under the Chinese Guidelines for the diagnosis and treatment of acute promyelocytic leukemia (2018) [9].

After completing induction chemotherapy, the blood cell counts returned to normal levels and the bone marrow aspirate showed hematological complete remission (HCR) but not major molecular remission (MMR) with the detection of *PML-RARA* by FISH and RT-PCR with the newly designed probes. Then, one cycle of the DA regimen (daunorubicin 60 mg/m2/d, d1-3; cytarabine 200 mg/m2/d, d1-7) and another cycle of the DA regimen (daunorubicin 45 mg/m2/d, d1-3; cytarabine 1 g/m2/12 h, d1-4) were administrated to the patient as consolidation chemotherapy. The patient is currently under MMR and HCR without any clinical symptoms or signs until now.

## Discussion and conclusion

APL is a unique subtype of acute myeloid leukemia and is characterized by a balanced reciprocal translocation between chromosomes 15 and 17. To the best of our knowledge, the ider(17)(q10)t(15;17)(q22;q12) has been found in approximately 1% of the reported secondary cytogenetic abnormalities in APL patients, which has been reported only in 85 APL cases worldwide [1, 3]. Here, we described a rare APL case with a false negative RT-PCR result and a *TET2* SNP rs2454206 that had ider(17)(q10)t(15;17)(q22;q12).

SNP rs2454206 is common in the *TET2* gene, which plays a role in epigenetic regulation of myelopoiesis. Wang *et al* reported that *TET2* SNP rs2454206AG/GG is associated with improved overall survival and event-free survival in AML patients with intermediate-risk cytogenetics features [10,11]. To the best of our knowledge, this mutation has not been reported in APL with ider (17)(q10)t(15;17)(q22;q12) and its prognostic significance in APL remains unclear.

RT-PCR is considered the most sensitive tool for diagnosing *PML-RARA*-positive leukemia. However, the false negative rate of RT-PCR for diagnosing *PML-RARA* is approximately 1% [9]. To the best of our knowledge, this is the first case report of a false negative for diagnosing *PML-RARA* in a patient that had ider(17)(q10)t(15;17)(q22;q12). In our experience, it is not advisable to rely only on RT-PCR for the diagnosis of *PML-RARA*-positive APL and FISH analysis was equivalent to or even better than RT-PCR. Although FISH is a highly specific confirmatory test for the diagnosis of APL, it cannot easily analyze low-level signal patterns, such as 2-5%, to determine the clinical significance of the results when used for the detection of minimal residual disease [12]. Therefore, RT-PCR and FISH are recommended to be performed as parallel tests for the diagnosis of APL.

The frequency of the clone with ider(17)(q10)t(15;17)(q22;q12) was higher than that of the clone with t(15;17) alone, which is consistent with the findings of other studies. Manola *et al* suggest that the duplication of der(17q), overrepresentation of the *RARA-PML* fusion gene and loss of the normal tumor suppressor gene *P53* mapped on 17p13 due to the loss of 17p, which plays an important role in oncogenesis and serves as a poor prognostic factor in leukemia, may contribute to this growth advantage [13].

Some reports suggested that ider(17)(q10)t(15;17)(q22;q12) might be a poor prognostic factor in APL [1,3,12–15]. Therefore, some reports showed that the clinical course of patients with ider(17)(q10)t(15;17)(q22;q12) did not seem to differ from that of the typical t(15;17)(q22;q12) [13,15]. The role of ider(17)(q10)t(15;17)(q22;q12) in APL is still an ongoing investigation and is limited by the small number of published cases. In our experience, the clinical outcomes of this patient may be due to the following 3 points. First, this patient was classified in the low-risk group at the initial diagnosis stage according to the 2017 NCCN guideline for AML, which correlates with the therapeutic effects, clinical outcomes and relapse for APL. Second, the early usage of arsenic trioxide combined with ATRA, has emerged as the new standard of care for patients with low-to-intermediate risk APL [16]. Last, continuous transfusion of platelet concentrates and fresh frozen plasma was conducted to maintain PLT ≥30×10^9^/L and FIB>1500 mg/L. APL is now considered to be the most curable subtype of acute myeloid leukemia in adults. Nevertheless, APL remains associated with a significant incidence of early death related to the characteristic bleeding diathesis [16]. Therefore, we infer that the patient reported here may benefit from vigorous supportive treatment during the combination of ATRA and arsenic trioxide in induction chemotherapy. However, more case reports and systematic analyses are needed to help us better understand the clinical, cytogenetic and molecular features and prognostic significance of ider(17)(q10)t(15;17)(q22;q12) in APL patients.

## Data Availability

The data sets used and/or analyzed during the current study are available from the corresponding author on reasonable request. All authors read and approved the final manuscript.

## Abbreviations

*PML*: promyelocytic leukemia gene
*RARA*: retinoic acid alpha gene
AML: acute myelocytic leukemia
APL: acute promyelocytic leukemia
FISH: fluorescence in situ hybridization
HCR: hematological complete remission
MMR: major molecular remission RT-PCR: reverse transcriptase-polymerase chain reaction
SNP: single nucleotide polymorphism
